# Delegates of the Ohio Dental College Association

**Published:** 1859-07

**Authors:** B. Bonsall, J. Taft


### DELFGATES OF THE OHIO DENTAL COLLEGE ASSOCIATION.

AT a called meeting of the Ohio Dental College Association, June 22d, 1859, the following members were appointed delegates to meet at Niagara, in accordance with the call published in the last number of the *Register:*

Drs. H. A. Smith, Jas. Richardson, J. T. Toland, J. Taft, Cincinnati, O.; A. Berry, Raymond, Miss.; G. W. Keely, Oxford, O.; Geo. Watt, Xenia, O.; J. S. King, Zanesville, O.; W. B. Webster, Richmond, Ind.; J. P. Melroy, Lawrance, Ind.; C. B. Chapman, Madison, Wis

On motion of Dr. Watt, it was

*Resolved*, That the delegates have power to fill vacancies in their number, from any members of this Association who may be present.

On motion, adjourned. B. BONSALL, Pres't.

J. TAFT, Sec'y.

